# Exploring affordability and healthcare-seeking behaviour for delivery and antenatal care among the poor and ethnic minorities in rural Northwestern Viet Nam

**DOI:** 10.1080/16549716.2018.1556573

**Published:** 2019-01-14

**Authors:** Chieko Matsubara, Tai Anh Nguyen, Hitoshi Murakami

**Affiliations:** a Bureau of International Medical Cooperation, National Center for Global Health and Medicine, Tokyo, Japan; b Center for Community Development Aid, Hoa Binh, Hoa Binh province, Viet Nam

**Keywords:** Health insurance, Viet Nam, out-of-pocket payment, preference of maternity care institution, ethnic minorities, the poor, private clinic, household income quartiles

## Abstract

**Background**: Health insurance (HI) has been introduced to achieve universal health coverage. In Viet Nam, mandatory HI for the poor and the minorities has been strengthened since 2012.

**Objective**: The study explored affordability and healthcare-seeking behaviour for delivery and antenatal care (ANC) among the poor and ethnic minority women after HI-reform in rural Northwestern Viet Nam.

**Methods**: A cross-sectional study was conducted in 2014 in Luong Son District, where the ethnic Muong live. Stratified simple random sampling was used to select 315 participants who had delivered a baby in the previous year.

**Results**: The HI coverage was 72.7% (229/315) and 30.9% of the mothers were living on less than USD 1.25 per household person per day. HI enrolment was predicted by ethnic minority status (Muong, aOR 18.3, 95% CI 6.4–52.6), rather than the household income. More than 80% of majority and minority respondents selected the institution by their trust in the quality of its care.

The institutional delivery was 100%, irrespective of HI status. The out-of-pocket expenses for normal delivery were significantly smaller for the insured than the uninsured (p < 0.001). The total cost of normal delivery proved to be a catastrophic payment (households spending > 5% of annual household income) for 17.6% and 31.7% of the insured and uninsured, respectively. The average number of ANC visits was more than four times for all quartiles, irrespective of the mothers’ HI status; however, all quartiles demonstrated more frequent visits to private clinics than commune health centres (public facility).

**Conclusions**: The results indicated that Vietnamese HI reform reduced the economic burden for both the poor and ethnic minorities in rural villages. However, further HI reforms should consider ways to reduce the catastrophic payments, fix the role of private facilities for appropriate resource mobilisation, and enhance the move towards universal health coverage.

## Background

Universal health coverage (UHC) has been one of most well-discussed targets in the context of the global health-related goals. The Sustainable Development Goal 3 (SDG3), target 8 is a call for action by United Nations for countries to strive for UHC by 2030 [1,2]. Facing financial barriers in accessing health care is one of the important issues in ensuring equity and responsiveness in health systems for the vulnerable and marginalised population. Therefore, financial protection is one of the key dimensions of the UHC, as well as the essential health service coverage [3]. Many efforts have been made, such as expanding health insurance (HI); however, over 800 million people spent more than 10% of their household budgets to pay for health care in 2017 [1].

In Viet Nam, HI coverage has been vigorously expanded since its initial implementation in 1992. The two major HIs in Viet Nam are compulsory HI (CHI) and voluntary HI (VHI). The former is primarily targeted at civil servants and formal sector employees. The VHI is targeted at self-employed, informal sector workers, and dependants of the CHI members [4].

The CHI seemed to play a critical role in ensuring HI coverage. The Health Care Funds for the Poor (HCFP) was incorporated into general CHI; the tax-based enrolment of the poor and near-poor to provide them with financial health protection represented a significant and judicious attempt by the government [4]. From the perspective of financial protection of the vulnerable and marginalised population, the CHI also incorporates tax-based enrolment of ethnic minorities and children aged six years or less [4]; thus, the vulnerable groups were mostly covered by the CHI. The Viet Nam Social Security (VSS) regularly reviewed the eligibility of the population for tax-based enrolment. Some people, such as the poor or near-poor, who were classified as vulnerable, were not allowed the option of participation and the VSS automatically enrolled them on the basis of their vulnerability status.

Despite these efforts that extend HI coverage to a larger fraction of the marginalised population in Viet Nam, a study in 2018 reported that OOP accounted for 56% of total health spending [5], and 1.3–1.7% of the incidence of impoverishing health spending based on the USD 3.10 per day limit [6]. Moreover, most beneficiaries are located in urban areas and formal sectors [7].

As regards the impact of HI on care-seeking, household surveys conducted in Hai Duong and Bac Giang provinces both indicated an increase in outpatient and inpatient utilisation rate among residents who were covered by HI, compared with those who were not [8]. However, the patients in the two highest quintiles used health services more than the patients in the lowest quintile in the Bavi district of Hatay province in 2001 and 2002 [9]. Further, household OOP health expenditure has been reported to be 50–70% higher [9,10]. Another study reported that, in rural areas, the risk of not delivering in a health facility among ethnic minority women, which became increased from five times that faced by majority women in 2006 to 20 times in 2010–2011 [11].

The objective of this study was to explore affordability and healthcare-seeking behaviour for delivery and antenatal care under the HI reform, particularly among the poor and the ethnic minorities, in the Luong Son district, which is a rural district in Hoa Binh province, of Northwestern Viet Nam.

## Methods

### Study setting

This study was conducted at the Luong Son district, one of the 10 districts of Hoa Binh province, which is located to the southwest of Hanoi, the capital of Viet Nam. The total population of the district in January 2014 was 97,446 [12]. Minorities, such as Muong, form a majority of the population in Hoa Binh province; comprised 63.9% of the total Hoa Binh population in 2009 [13].

### Study design and data collection

A cross-sectional study was conducted with stratified single-step cluster sampling method. Before data collection in December 2013, all 322 villages in Luong Son District were stratified into three groups. The first group was villages located in the town areas of the district, which are close to the district hospital (zone 1). Then, among villages located in commune (sub-district) areas, villages located close to commune health centre (CHC) were placed in the second group (zone 2) and villages which were far from both the district hospital and the CHC were placed in the third group (zone 3). From each group, seven villages were randomly selected in accordance with their population size using probability proportionate to size (PPS) sampling to minimise sampling bias, as the number of mothers were supposed to be larger in zone 1 & 2 than in zone 3. In total, 21 villages were selected. The average distance from CHC and the district hospital in each zone was 2.9 km and 3.9 km, respectively in zone 1; the values were 1.0 km and 24.8 km in zone 2, and 3.0 km and 26.8 km in zone 3.

Over three weeks in January 2014, we interviewed all mothers who delivered their last baby in the previous year (2013) in the 21 villages selected. We excluded mothers whose children were not alive at the time of the study, considering the state of mothers who had lost their child. In total, 315 mothers were interviewed, and no mother declined participation in this study.

We asked questions about their HI coverage, socio-demographics, healthcare-seeking behaviours, and payment made at the time of their delivery. The participants were divided into four quartiles, based on the household annual income (Annual household income quartile). The out-of-pocket (OOP) payment of the insured and the uninsured groups were compared by *t*-test for both normal delivery and caesarean section. The households whose total cost for delivery more than 5% of their household annual income were deemed to have made a catastrophic payment. Logistic regression models were constructed with the forced entry method by considering ‘status of HI’ as the dependent variable, and antenatal care (ANC) equal to or more than four visits (ANC ≥ 4), household annual income quartile, ethnicity, mode of delivery (normal trans-vaginal or caesarean), final education level, and occupation as the independent variables. In this study, ANC ≥ 4 was applied even though WHO has recommended ANC ≥ 8 since November 2016 [14]. This was because the World Health Organization Western Pacific Region reported that no country had applied ANC ≥ 8 as on November 2017, and it itself applied ANC ≥ 4 in its review of maternal health care in 2018 [15].

### Data analysis

Data were analysed by using SPSS statistics version 21 for Windows (IBM Corp, Armonk, NY). Statistical significance was set at *p* < 0.05.

## Results

The HI coverage was 72.7% (229/315) in this study in 2014 in the Luong Son District, Hoa Binh province. The HI coverage includes the coverage by either CHI or VHI.

Table 1 summarises the basic characteristics of the mothers who were interviewed. Majority of the study population belonged to ethnic minorities (Muong, 196, 63%), rather than the Kinh (Viet), who are the majority all over the whole country. More Muong were covered by HI than Kinh. High school (129, 42%) was the most common highest educational level and farming (149, 61%) was the most common occupation. Further, there was no statistically significant difference in HI coverage among the income quartiles.

**Table 1.** Socio-demographic status of the mothers who had deliveries in 2013 in the Luong Son district, Hoa Binh province, Viet Nam (n = 315).

	Health insurance enrolment				
	Yes		No		
	n	(%)	n	(%)	*p*-value
Ethnicity (Missing = 3)					
Kinh (Viet)	60	(51.7)	56	(48.3)	**<0.001**
Minority	167	(85.2)	29	(14.8)	
Household annual income (Missing = 2)					
Lowest quartile	70	(74.5)	24	(25.5)	0.17
2nd quartile	58	(65.9)	30	(34.1)	
3rd quartile	47	(72.3)	18	(27.7)	
Highest quartile	54	(81.8)	12	(18.2)	
Age					
(mean, yrs)	(27.2)		(27.0)		0.22
Education (Missing = 10)					
Primary	12	(70.6)	5	(29.4)	0.19
Secondary	61	(70.1)	26	(29.9)	
High school	90	(69.8)	39	(30.2)	
University	59	(83.1)	12	(16.9)	
Occupation (Missing = 68)					
Farmer	111	(74.5)	38	(25.5)	**<0.001**
Formal sector/Industrial worker	33	(86.8)	5	(13.2)	
Shop/Restaurant	2	(20.0)	8	(80.0)	
Civil servant	47	(94.0)	2	(6.0)	
Zone					
Zone 1 (near district hospital)	70	(63.6)	40	(36.4)	**0.003**
Zone 2 (near CHC)	86	(84.3)	16	(15.7)	
Zone 3 (no health facility)	74	(71.8)	29	(28.2)	
Total number of ANC (Missing = 1)					
(mean, times)	(6.3)			(5.7)	0.15
0 to 3 times	53	(75.7)	17	(24.3)	0.55
≥4 times	176	(72.1)	68	(27.9)	
Place of ANC (Missing = 1)					
CHC (mean, times)	(2.2)		(1.7)		**0.006**
District hospital (mean, times)	(0.5)		(0.2)		**0.028**
Provincial hospital (mean, times)	(0.2)		(0.2)		1.00
National hospital (mean, times)	(0.4)		(0.2)		0.18
Private clinic (mean, times)	(3.0)		(3.4)		0.33
Place of Delivery (Missing = 3)					
Home	0		0		
CHC	15	(75.0)	5	(25.0)	0.53
District hospital	152	(71.7)	60	(28.3)	
Provincial hospital	32	(78.0)	9	(22.0)	
National hospital	18	(75.0)	6	(25.0)	
Private clinic	10	(66.7)	5	(33.3)	
Caesarean delivery (Missing = 5)					
Yes	50	(71.4)	20	(27.4)	0.81
No	175	(72.9)	65	(27.1)	

Abbreviations: CHC, Commune Health Centre; ANC, Antenatal Care.

As regards ANC visits, as presented in Table 1, there was no statistically significant difference between ANC≥4 and ANC<4 among the insured and uninsured (p-value, 0.55); ANC≥4 was recommended by the Vietnamese health authority at the time of data collection (2013). Further, although not presented in Table 1, 97 (83%) Kinh mothers and 114 (74%) Muong mothers had ANC ≥4; the difference in visits between the Kinh and Muong was not statistically significant (*p*-value, 0.065).

All 315 participants opted for institutional delivery, irrespective of their HI status. In addition, the HI enrolment did not indicate any statistical association with the proportion of caesarean sections conducted (*p*-value, 0.81).

The median number of household members was four (IQR = 4–6) and the median household annual income was Viet Nam Dong (VND) 60,000,000, which was equivalent to USD 2,644 as of March, 2017 (IQR = VND 48,000,000–96,000,000). Further, 97 (30.9%) of the mothers were living on less than USD 1.25 per person per day. Hence, they were regarded as being in absolute poverty. However, there was no statistically significant difference between the HI enrolment and the household annual income quartiles (*p*-value, 0.17).

Table 2 indicates the logistic regression analysis of factors associated with HI enrolment. Only residential zone (near urban area [near CHC), aOR 10.5, 95% CI 3.1–35.3); rural area without health facility, aOR 4.7, 95% CI 1.7–13.3), ethnic minorities, such as Muong (aOR 18.3, 95% CI 6.4–52.6), mothers’ final education level (university or higher, aOR 28.6, 95% CI 2.9–281.7), and occupation (formal worker/industrial worker, aOR 8.2, 95% CI 2.0–34.0 with farmers as reference) indicated statistically significant association with the status of HI enrolment after adjusting for other independent variables (*p*-value, <0.05). HI coverage did not indicate any significant association with ANC ≥ 4 and quarterly household annual income.

**Table 2.** Logistic regression analysis of factors associated with health insurance coverage in the Luong Son district, Hoa Binh province, Viet Nam in 2013.

		Health insurance coverage (n = 284)				
		B	S.E.	*p*-value	AOR	95%CI
Zone						
Zone 1 (near district hospital)	Ref.					
Zone 2 (near CHC)		2.35	0.62	**<0.001**	10.45	(3.10–35.27)
Zone 3 (no health facility)		1.56	0.53	**0.003**	4.72	(1.68–13.28)
Age		0.04	0.05	0.42	1.04	(0.95–1.13)
Education						
Elementary	Ref.					
Junior High		0.74	0.82	0.37	2.10	(0.42–10.45)
High School		1.36	0.89	0.13	3.91	(0.68–22.41)
University or higher		3.35	1.17	**0.004**	28.55	(2.89–281.69)
Occupation						
Farmer	Ref.					
Formal sector/Industrial worker		2.10	0.73	**0.004**	8.19	(1.98–33.96)
Shop/Restaurant owner or worker		−1.26	1.21	0.30	0.28	(0.03–3.05)
Civil servant		1.82	1.05	0.08	6.15	(0.79–47.91)
Household annual income						
Lowest	Ref.					
2nd quartile		−0.37	0.53	0.48	0.69	(0.25–1.94)
3rd quartile		−0.46	0.66	0.48	0.63	(0.17–2.29)
Highest		0.53	0.82	0.51	1.71	(0.34–8.47)
Ethnicity						
Kinh (Viet)	Ref.					
Muong		2.91	0.54	**<0.001**	18.33	(6.38–52.62)
Caesarean section						
No	Ref.					
Yes		−0.60	0.52	0.25	0.55	(0.20–1.52)
ANC 4 ≥ times						
No	Ref.					
Yes		−0.16	0.47	0.81	0.89	(0.36–2.23)

Cox-Snell R^2^ = 0.30; Nagelkerke R^2^ = 0.46.

Abbreviations: SE, Standard Error; AOR, Adjusted Odds Ratio; CI, Confidence Interval; CHC, Commune Health Center; ANC, Antenatal Care. Missing = 28.

**Figure 1.**
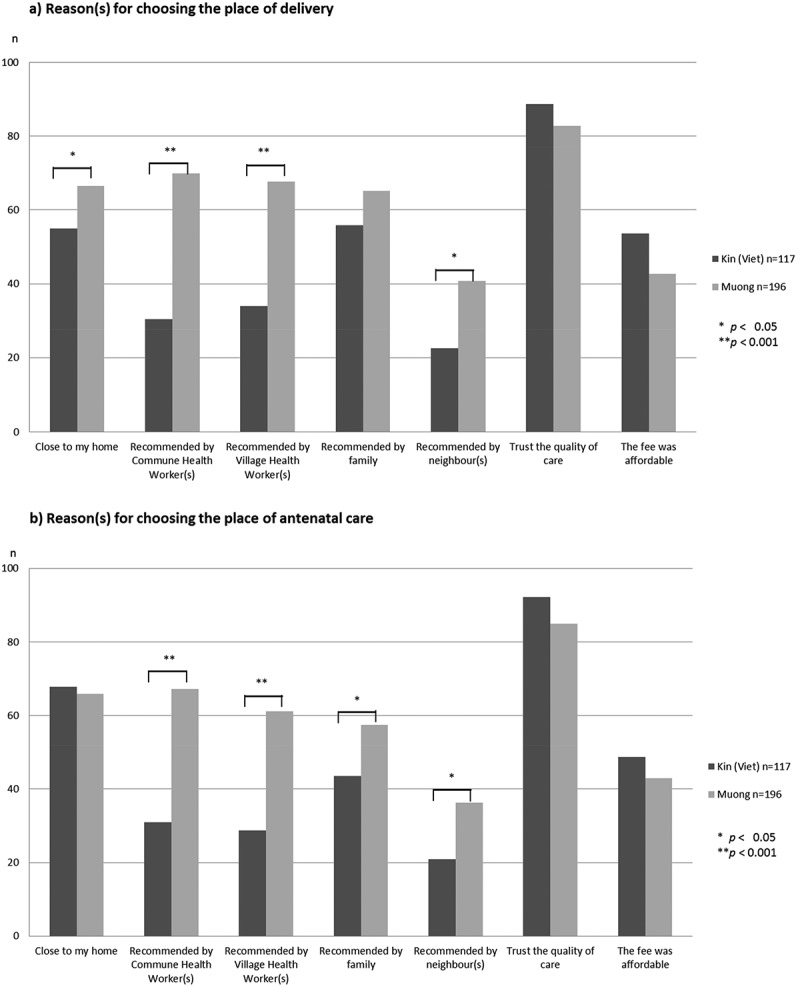
Reason(s) for choosing the place of delivery and antenatal care by Kinh (Viet, Majority) and Muong (Minority) in the Luong Son district, Vietnam in 2014 (Multiple answers).

Figure 1 indicates the reasons for choice of facility as the place of delivery (1-a) and antenatal care (1-b) among the ethnic groups. The mothers were asked to choose one or more responses from multiple options, as long as they applied. More than 80% Kinh and Muong mothers chose a facility based on their trust in the quality of care. Further, compared to the Kinh, more Muong mothers chose a facility based on recommendation by commune health workers or village health volunteers or neighbours and the observations were statistically significant. There was no statistically significant difference between Muong and Kinh in the choice of facility for ANC. However, more Kinh mothers chose a facility close to home or delivery than Muong mothers (*p*-value <0.05).

Table 3 presents OOP (median) in normal delivery and caesarean section delivery by household annual income quartiles and HI status. Households covered by HI indicated lower OOP in all quartiles, including the poorest quartile. Moreover, households covered by HI indicated lower OOP, with a statistically significant difference for all quartiles except the richest one. In the case of caesarean section delivery, households covered by HI indicated lower OOP in all household income quartiles, including the poorest quartile. However, the difference was statistically significant only for the second and third income quartiles.

**Table 3.** Median payment to health facility for normal delivery (n = 234) and caesarean section delivery (n = 66) among mothers, with and without health insurance, who delivered in the Luong Son district, Hoa Binh province, Viet Nam, in 2013 by household annual income quartile.

		Health insurance			
		Insured		Uninsured	
Household annual income quartile	*p*-value	n	Median (VND)	n	Median (VND)
Normal delivery (n = 234)					
Lowest	**< 0.001**	37	150,000	19	900,000
2nd quartile	**0.01**	56	200,000	21	980,000
3rd quartile	**0.006**	44	250,000	14	1,200,000
Highest	0.07	37	265,000	6	735,000
Caesarean section delivery (n = 66)					
Lowest	0.15	9	650,000	4	2,500,000
2nd quartile	**0.013**	8	1,650,000	6	4,850,000
3rd quartile	**0.004**	12	775,000	8	3,050,000
Highest	0.77	13	1,550,000	6	2,950,000

Abbreviation: VND, Vietnamese Dong. USD 1 = VND 22,692 VND as of March, 2017.

Aside from the payment to health facility, medical expenditure for the delivery care consisted of transportation cost and other non-medical expenditures, such as hospital meals fee, as shown in Table 4. Table 4 showed that the rate of catastrophic payments (> 5%) incurred on normal delivery among mothers with HI and without HI were 17.6% and 31.7%, respectively; the rates on by caesarean section delivery among mothers, with HI and without HI were 51.1% and 80.0%, respectively.

**Table 4.** Rate of catastrophic payment caused by normal delivery and caesarean section delivery among mothers, with and without health insurance, who delivered in the Luong Son district, Hoa Binh province, Viet Nam in 2013.

		Payment to health facility	Transportation cost	Other non-medical expenditure	Catastrophic payment^§^	
	n	Median (VND)	Median (VND)	Median (VND)	>5% (%)	>10% (%)
Normal delivery						
Insured	165	200,000	150,000	200,000	17.6	6.7
Uninsured	60	1,000,000	200,000	300,000	31.7	10.0
Caesarean delivery						
Insured	47	850,000	650,000	1,025,000	51.1	19.5
Uninsured	20	3,050,000	550,000	1,500,000	80.0	45.0

§Catastrophic payment >5% and >10% means the % of households whose total cost for delivery was more than 5% and 10% of annual household income, respectively.

Abbreviation: VND, Vietnamese Dong. USD 1 = VND 22,692 VND as of March, 2017.

**Figure 2.**
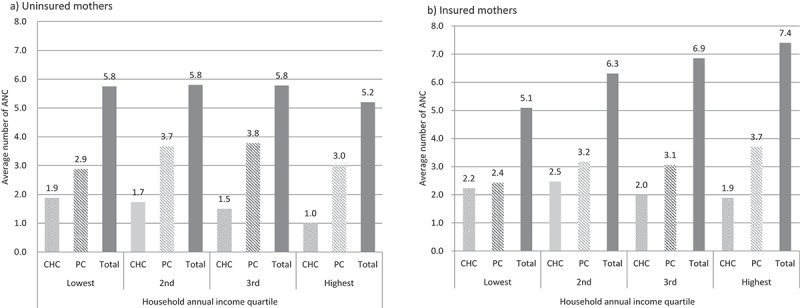
Average number of antenatal care (ANC) visits by place and household annual income quartile, with and without health insurance, in the Luong Son district of Hoa Binh province, Viet Nam in 2014. Abbreviation: ANC, Antenatal care; CHC, Commune health centre; PC, Private clinic Abbreviation: CHC: ANC, Antenatal care; Commune health centre, PC: Private clinic

Concerning ANC, Figure 2 shows that the average number of ANC visits were more than four (ANC ≥ 4) in all household economic quartiles among both the insured mothers (2-a) and the uninsured ones (2-b). There were mothers who received ANC at venues other than CHC and PC, such as the district, the provincial and the national hospitals. However, since the number of ANCs at these other places was very small, we did not show them in Figure 2. However, the total number of ANCs included the number of ANCs at the other places. The insured visited CHC more often than the uninsured across all income quartiles. However, mothers in all quartiles demonstrated less frequent visits to a CHC than private clinic, despite the latter not being covered by HI. Out of the total mothers (315), 239 (76%) went to a private clinic for their ANC at least once (not presented in the table or figure).

## Discussion

The study indicated that the Vietnamese policy to expand the HI coverage to the poor and minorities worked well in rural Northwestern Viet Nam. Further, for both normal delivery and caesarean section, the average cost of delivery was lower in the HI group as compared to the non-HI group for all quartiles of the household annual income. In particular, in a normal delivery, all quartiles except the richest revealed a lower delivery cost with a statistical significance in the HI group, compared with the non-HI group. The logistic models demonstrated that the poor and ethnic minorities were not factors influencing HI enrolment and ANC ≥4. The Vietnamese HI system supported enrolled mothers and their family; thus, the poor and ethnic minorities are highly protected against excessive expenditure during child delivery.

The result of the study also indicated that the HI coverage among the poor mothers who delivered in 2013 was comparable and even better than the non-poor and ethnic minorities in the Luong Son district, in rural Northwestern Viet Nam. Such equitable coverage contributed to a lower amount of OOP payment to health facility for normal and caesarean section delivery across all income quartile among the insured. The reduction in OOP was consistent with previous studies conducted in Viet Nam [16,17]. Further, ethnic minorities group had significantly higher HI coverage than the majority Kinh population. This is due to compulsory enrolment of ethnic minorities living outside urban areas. However, there is a need to find ways to expand VHI coverage among the non-vulnerable population, even though that would be a difficult task.

HI’s suppression of delivery care costs has also been reported by previous studies in Senegal [18] and China [19]. A public-private partnership financing scheme in the state of Gujarat in India [20] and universal delivery exemption in Ghana [21] also had the same effect. The studies indicate that the cost of suppression of HI does not only apply to maternal health care costs but to overall health costs. In Rwanda and Gujarat, community-based insurance protected households from catastrophic health spending [22,23]. In Egypt, a school-based HI reduced OOP health expenditure [24].

However, it should be noted that the financial support by HI might not be sufficient to prevent catastrophic payment in the study area. A recent study pointed out that the Northwestern mountainous region had one of the highest poverty areas in Viet Nam [25]. This study found that the rate of catastrophic payment by households (> 5% of annual income) with HI for normal delivery and caesarean section delivery were 17.6% and 51.1%, respectively. Non-disbursed costs, such as transportation and other non-medical costs, were also incurred by a significant proportion of the households in this study. Similarly, transportation cost accounted for almost half of the total expenditure on a normal delivery in Tanzania [26,27] and Nepal [28]. As for non-medical costs, hospital meal, and delivery kit fees were the common ones in our study. Unofficial provider payment was reported to be associated with ‘free’ delivery care in Tanzania [29]. The transportation and non-medical costs may lead to financial hardship for very poor households even after they have been covered by HI.

This research indicated that distance from the district hospital (downtown of the district), ethnic minority status, higher education level of mothers, and occupations (formal sector/industrial workers) were significantly associated with HI enrolment. A previous study in Ghana [30] had also indicated that educational status of women was a strong determinant of their HI enrolment. In another study conducted in the USA [31], employment and job characteristics were key determinants of disparities in access to HI. Thus, these studies, including the current one, indicate that education and occupation are the key social factors determining HI coverage. Regarding ethnicity, the difference in health care utilisation across different ethnic groups in the USA, particularly between Hispanics/African Americans and whites, was attributable to insurance coverage [32,33]. Conversely, at our study site, the ethnic minority status was associated with higher HI coverage. The result reflects the success of the HI policy reform in Viet Nam to ensure favourable HI coverage among the remote and ethnic minority population owing to affirmative enrolment of such minorities by providing government subsidy.

For care-seeking, service utilisation for both delivery and ANC were not significantly different after HI coverage. First, the institutional delivery rate was 100%, irrespective of their HI status, mainly because of the strong promotion of such a delivery and the discouragement of home delivery by the health authority; however, facility selection criteria varied across between ethnic groups. Different studies around the world identified different factors, such as economic status, education, empowerment [34,35], age, parity, walking distance to health facility [36,37], HI coverage, ethnicity [38] and ANC attendance [39], as contributing to institutional delivery. The universal institutional delivery, demonstrated in the Luong Son district, is remarkable, because there are various barriers that may hinder mothers from delivering at health facilities. HI did not increase the proportion of mothers who underwent caesarean section in our study site, unlike the situation in Shanghai, China [40] where HI was associated with a higher caesarean rate.

The average total number of ANC visits were more than four, irrespective of the household economic status and the HI status. In addition, the total number of ANC visits increased according to the rise in the household economic status of the insured mother. Conversely, this increase was not found among non-insured mothers. There are many studies which also revealed that HI increased the ANC attendance, both in Viet Nam [16] and in African countries [18]. Dissociation between HI coverage and the number of ANC visits in our study can partially be explained by the role of private clinics. These private clinics were, in most cases, not contracted with the HI. The anecdotal finding suggests that mothers go to private clinics to witness a baby’s ultrasonic three dimension images, which are not covered by HI. This study indicated that even mothers in the lowest quartiles went to private clinics for their ANC, irrespective of their HI status. This might be explained by the reason(s) for choosing the ANC venue; mothers chose one according to the quality of care provided. However, this suggests that a poor family would have incurred further costs on the ANC received at the private clinic. In the future, it may also be necessary to extend/reconsider insurance benefits (coverage) according to patient needs, such as contract with private clinics, to reduce more OOP for the poor.

## Methodological considerations

This study has the following limitations. First, the cross-sectional nature of the research means that it cannot identify causal relationships. The second limitation of this study is the possibility of recall bias. Given that costs were assessed based on verbal reporting of costs by the respondents without verifying receipts, there could be a possibility of over estimation or under estimation of costs. However, the recall period was short (one year), because we asked about the delivery during the previous year and an earlier study showed that mothers’ memories of their birth experiences were very accurate even after 15–20 years [41]. Third, we did not take into considerations the order of multiple reason(s) for choosing the place of delivery and antenatal care. Future studies would show the reason(s) more clearly if the order is considered in the multiple response(s). Fourth, this study did not assess caesarean section delivery by severity of complication since the emphasis was only on the affordability of the cost of treatment. However, we do acknowledge this as a limitation, given that there could be variations in cost and treatment-seeking between cases of moderate and severe complications.

## Conclusions

The following are the three main conclusions of the study. First, expansion of HI enrolment by the government was successful in covering the poor and ethnic minorities in the Luong Son district, in the predominantly rural Northwestern Viet Nam. Second, such equitable coverage contributed to lower amount of OOP at the health facility for normal delivery and caesarean section across all income quartiles by the insured than the uninsured. Third, HI supported payment to health facility for medical treatment; however, the households still face financial burden caused by transportation cost and non-medical payments in case the household income was low. Fourth, mothers-seeking healthcare go to a private clinic, when they think the service is necessary, irrespective of whether the HI benefit packaged covered it. In addition, further studies are needed on the expansion of VHI coverage among the non-vulnerable population; this requires further assessment of the factors associated with VHI enrolment.

The results of this study suggested that HI reform worked well in villages in reducing the economic burden for both ethnic minorities and the poor; however, further HI reforms are necessary to consider ways to prevent catastrophic health expenditure for the poor HI enrolees and clarify the role of private health facilities with the aim of more appropriate resource mobilisation for HI and facilitating the move towards universal health coverage.

## Data Availability

The datasets used and/or analysed during this study are available from the corresponding author on reasonable request.

## Paper context

Health insurance support has not yet been sufficient for the vulnerable in many low- and middle-income countries. This study seeks to explore affordability and healthcare-seeking behaviour for maternity care under the Vietnamese health insurance reform since 2012. The reform worked well in rural villages, but 17.6% of the insured spent more than 5% of their annual income on normal delivery. Most preferred private clinics over public institutions for antenatal care; this led to the incurring of more expenses.
